# Effects of Ocular Direct Current Stimulation on Full Field Electroretinogram

**DOI:** 10.3389/fnins.2021.606557

**Published:** 2021-02-16

**Authors:** Maren-Christina Blum, Benjamin Solf, Alexander Hunold, Sascha Klee

**Affiliations:** Institute of Biomedical Engineering and Informatics, Technische Universität Ilmenau, Ilmenau, Germany

**Keywords:** electroretinogram, full field ERG, ocular electrical stimulation, direct current stimulation, non-invasive brain stimulation, transorbital electrical stimulation

## Abstract

Studies on weak current stimulation (1–2 mA) examine effects on neuronal cells for the treatment of neurological diseases, like depression. Ocular current stimulation showed positive effects on retinal nerve cells which indicate that neurodegenerative ocular diseases, e.g., glaucoma, can be treated with current stimulation of the eye. However, up to now it remains unclear which exact retinal cells can be influenced. During an ocular direct current stimulation, a significant reduction of the characteristic P50 amplitude of a pattern-reversal electroretinogram (PERG) was found for an anodal and a cathodal stimulation. This current stimulation effect could originate from the modulation of pre-ganglion cell activity or by changes in local ON and OFF responses of ganglion cells. For clarification, we investigate acute direct current stimulation effects on a full field electroretinogram (ERG), which represents the activity of pre-ganglion cells (specifically cones and bipolar cells). The ERG from 15 subjects was evaluated before (ERG 1) and during (ERG 2) an ocular direct current stimulation with 800 μA over 5 min. The current was applied through a ring rubber electrode placed around the eye and a 25 cm^2^ rubber electrode placed at the ipsilateral temple. For ERG measurements, sintered Ag/AgCl skin-electrodes were positioned on the lower eyelid (active), the earlobe (reference), and the forehead (ground). The volunteers were stimulated in three independent sessions, each with a different current application (randomized order): cathodal polarity, anodal polarity (referred to the electrode around the eye), or sham stimulation. The changes between the two ERG measurements of the characteristic full field ERG amplitudes, a-wave, b-wave, and b′-wave (b-wave measured from zero line) were tested with the Wilcoxon signed-rank test (α = 0.05). Comparing before to during the current stimulation for all applications, the ERG waves showed no effects on amplitudes or latencies. Furthermore, no significant difference between the cathodal, anodal, and sham stimulation could be found by a Friedman test. These results indicate an unlikely contribution of pre-ganglion cells to the previously reported stimulation effect on PERG signals.

## Introduction

Research on the effects of weak current stimulation on neuronal cells in humans has been taking place for the treatment of neurological diseases, e.g., depression (Yokoi and Sumiyoshi, 2015; Lefaucheur et al., 2017). Effects on neuronal activity, such as the manipulation of visual evoked potentials (VEP) by direct current stimulation of the visual cortex, could be demonstrated. In these studies, a polarity-dependent influence (increasing or decreasing) on the characteristic VEP amplitudes was shown (Antal et al., 2004; Accornero et al., 2007; Ding et al., 2016; Wunder et al., 2018). Chow et al. (2004) reported vision improvements in patients with an implanted electric visual prosthesis even in retinal areas far from the prosthesis. These improvements were attributed to the weak current pulses applied by the prosthesis to the underlying retinal cells (Chow et al., 2004). Numerous animal studies and first pilot studies in humans on the modulation of retinal cell activity by ocular current stimulation have been performed. In rats, a weak biphasic current stimulation increased the survival of retinal ganglion cells as well as photoreceptors (Morimoto et al., 2005, 2007, 2012; Tagami et al., 2009; Schatz et al., 2012). In general, positive effects were found on the development, functionality, and stability of retinal nerve cells (Sehic et al., 2016). Human studies mainly investigated the effect of ocular current stimulation on neurodegenerative eye diseases such as glaucoma (Röck et al., 2014; Gil-Carrasco et al., 2018; Ota et al., 2018), retinitis pigmentosa (Schatz et al., 2011, 2017; Wagner et al., 2017; Jolly et al., 2020), Stargardt disease (Röck et al., 2013), macular degeneration (Shinoda et al., 2008; Anastassiou et al., 2013; Chaikin et al., 2015), retinal artery occlusions (Inomata et al., 2007; Naycheva et al., 2013), or optic neuropathy (Fedorov et al., 2011; Gall et al., 2011; Sabel et al., 2011). The relationship between stimulation parameters such as current intensity, frequency, or duration and the current effects on the retinal cells remained ambiguous (Sehic et al., 2016). Furthermore, it is unknown which retinal cells can be influenced in detail. In a preceding study (Blum et al., 2020), the authors attempted to address this research question, by examining the effects of an ocular direct current stimulation on the pattern-reversal electroretinogram (PERG). There, the characteristic P50 amplitude was significantly reduced during anodal or cathodal direct current stimulation, while no effect could be found for sham stimulation. It is noticeable that both current polarities led to a reduction of the amplitude in this study. On the contrary, transcranial direct current stimulations of the visual cortex demonstrated opposing effects on VEP, depending on the stimulation polarity (Antal et al., 2004; Accornero et al., 2007; Ding et al., 2016). Considering the PERG origin, two mechanisms could potentially explain the stimulation effect reducing the P50 amplitude independent of the current polarity, as Blum et al. (2020) introduced in their work. The PERG is composed of local ON and OFF responses, which cancel each other out. A PERG response is measurable because of small differences between ON and OFF responses in the intra-retinal calculation (Bach and Hoffmann, 2006). Therefore, one hypothesis is that the current stimulation influences the ON and OFF pathway differently, but the settlement within the retina always results in a reduction of the P50 amplitude and an unchanged N95 amplitude. The other mechanism refers to the cellular origin of the PERG amplitudes. The PERG P50 amplitude is influenced by ganglion cells and pre-ganglion cells, such as bipolar cells, amacrine cells, horizontal cells, rods, and cones, while the N95 amplitude originates from ganglion cells (Bach and Hoffmann, 2006). Thus, the polarity independence of the current stimulation effect could be explained by current stimulation influences on pre-ganglion cells but not on retinal ganglion cells. The functionality of pre-ganglion cells, especially photoreceptors and bipolar cells, can be examined in full field ERG, which is a standardized electrophysiological test (Frishman, 2006).

In order to advance the understanding of current stimulation effects on retinal cell types, this study focuses pre-ganglion cells by means of current stimulation effects on the full field ERG. The aim of the study was to analyze differences between the amplitudes and latencies before and during an ocular current stimulation. The current stimulation was performed with two current stimulation polarities (cathodal and anodal) and a sham condition for a controlled analysis of potential polarity-dependent effects. Based on the current stimulation effects on the photoreceptors in animal studies, and the PERG changes found in our previous study, we hypothesized, that the full field ERG would be affected by current stimulation. Following the above-mentioned current stimulation effects reported in VEP studies, we expected polarity-dependent current stimulation effects on the full field ERG. The results can contribute to the understanding of current stimulation effects on simultaneously recorded electrophysiological activity of retinal cells, specifically whether the effects found by Blum et al. (2020) are due to ganglion or pre-ganglion cell influence. Furthermore, the knowledge about cell types, that can be affected by current stimulation potentially indicates which neurodegenerative retinal diseases can benefit from therapy with ocular current stimulation.

## Materials and Methods

### Participants

Fifteen healthy subjects (mean age: 27.5 ± 4.5 years, 8 females) participated in the study, which was permitted by the Ethics commission at the medical faculty of the Friedrich-Schiller-University Jena, Germany. All volunteers were asked about their state of health and provided written informed consent according to the Declaration of Helsinki on biomedical research involving human subjects (Tokyo amendment). Exclusion criteria included the following: neurological, eye, skin or heart diseases; metal implants in the head area; allergies or hypersensitivities of the skin; pregnancy; refractive error >±2 diopter. In total, the volunteers were invited to three independent sessions, in each of which a different current application (i.e., cathodal polarity, anodal polarity or sham stimulation) was performed in randomized order. All measurements were conducted by the same individual.

### Measurement Setup

The full field flash stimulation was performed monocularly using an electrophysiological full field stimulator (RETI-port/scan 21 Q450 stimulator, Roland Consult Stasche & Finger GmbH, Brandenburg a.d. Havel, Germany). The subject had to look at a fixed red point in the center of the stimulator and the head was placed in a height-adjustable chin rest.

A Cubias-M amplifier system (neuroCare Group GmbH, Munich, Germany) recorded the ERG with a dynamic range of ± 170 mV, a 24-bit analog-to-digital converter, an input impedance of ≥10 GΩ, an internal noise level of ≤0.9 μV, and a sampling rate of 2,000 sps. Sintered Ag/AgCl ring electrodes (EASYCAP GmbH, Herrsching, Germany) were used to detect the ERG. The active electrode was placed on the lower eyelid, while the reference electrode was attached to the ipsilateral earlobe and the ground electrode was placed on the forehead of the volunteer. In order to ensure good signal quality, the skin at the electrode positions was prepared with NuPrep contact-gel (Weaver and Company, Aurora, CO, United States) and the electrodes were coated with Ten-20 conductive EEG paste (Weaver and Company, Aurora, CO, United States). Further, the electrodes were fixed with tape.

Direct current was applied using a DC-Stimulator MC (neuroCare Group GmbH, Munich, Germany) that was powered by a battery extension. A ring rubber electrode (outer/inner diameter: 75 mm/30 mm; thickness: 2 mm) was used in combination with Ten-20 conductive gel to feed current into the eye. The rubber electrode had a cutout in the area of the lower eyelid to allow the placement of the ERG recording electrode. The counter rubber electrode (25 cm^2^, thickness: 2 mm) was placed in a saline-soaked (10 ml) sponge and positioned at the ipsilateral tempus with a fixation strap. The volunteer’s hair was lightly moistened with saline solution to achieve a low electrode impedance before applying the counter electrode.

**FIGURE 1:**
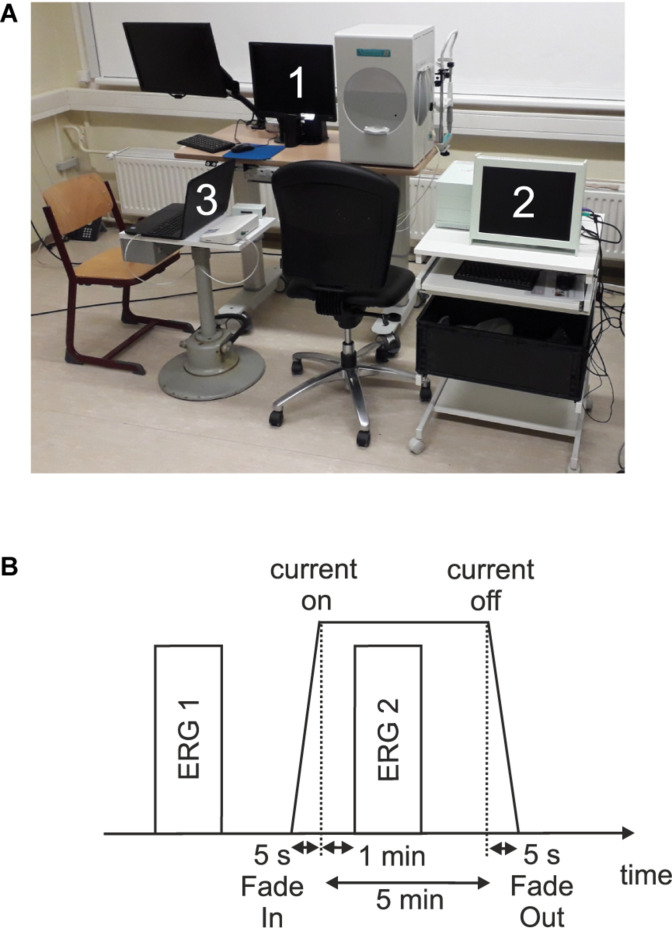
**(A)** Measurement setup which consists of (1) a visual stimulator system (RETI-port/scan 21 Q4; Roland Consult Stasche & Finger GmbH, Brandenburg a.d. Havel, Germany), (2) the current stimulator system (MC-stimulator DC; neuroCare Group GmbH, Munich, Germany), and (3) the amplifier system (Cubias-M; neuroCare Group GmbH, Munich, Germany). **(B)** Measurement timeline.

Figure 1A shows an overview of the measurement setup.

### Stimulation Parameters

The full field visual stimulus was a white flash (≤5 ms) with a strength of 3 cds/m^2^ and no background illumination. In total, 250 single flashes per measurement were presented at 2 Hz. During an ERG measurement, the examination room was darkened, while between the measurements it was lightened again.

The current stimulation was performed with a direct current of 800 μA over a duration time of 5 min. The current intensity was chosen to be higher than the mean phosphene threshold of healthy subjects (Freitag et al., 2019). In order to avoid skin irritation under stimulation electrodes and transient current sensation, the current was linearly ramped on (Fade-In) over 5 s at the beginning of the current stimulation and was ramped down linearly (Fade-Out) for 5 s at the end of the stimulation. There were three possible current applications: cathodal polarity, anodal polarity or sham stimulation. The applied current polarity (cathodal and anodal) refers to the stimulation electrode around the eye. Sham stimulation was performed so that no current flow was generated at the electrodes. The subjects were only informed that a current stimulation was performed.

### Experimental Timeline

In each of the three sessions, two single ERG recordings were performed: one before (ERG 1) and one during (ERG 2) the current stimulation. Figure 1B shows a measurement timeline. After preparing and attaching the electrodes, an impedance test of the ERG electrodes was performed. Here, impedances ≤15 kΩ and a difference ≤5 kΩ between the electrode impedances were admitted. The ERG 1 measurement represented the baseline. Subsequently, an impedance test (sinus alternating current, 200 μA, 20 Hz) for the current stimulation electrodes was carried out. In order to start the current stimulation, the impedance had to be ≤8 kΩ and subsequently the current stimulation was executed. One minute after the start of the current stimulation, the ERG 2 measurement was performed.

### Signal Processing

Signal processing was performed with MATLAB, version 2018b (The Mathworks, Inc., Natick, MA, United States). The ERG raw signal was filtered forward and backward to avoid phase shifting with an infinite impulse response (IIR) high pass (Butterworth; filter order: 3; half power frequency: 0.75 Hz) and low pass (Butterworth; filter order: 10; half power frequency: 70 Hz) filter. The sweeps that contained amplitudes higher than 100 μV after the filtering process were evaluated as artifact afflicted. For the remaining sweeps, for each sweep, the Pearson correlation was calculated to the mean over all remaining sweeps, and 200 sweeps with the highest correlation coefficient were averaged for each subject. The averaged signal was centered at the time point zero to the amplitude zero. The a-wave amplitude was defined as the first minimum of the averaged ERG measured from the zero line. The b-wave amplitude was determined as the first maximum measured from the a-wave minimum (McCulloch et al., 2015). Moreover, the b′-wave amplitude measured from zero line to the maximum b-wave peak was analyzed.

### Analysis

Statistical analysis was performed using IBM SPSS Statistics, version 25 (IBM Corp., Armonk, NY, United States). The significance level was set to α = 0.05. The normal distribution hypothesis was rejected by the Shapiro–Wilk test.

The primary aim of the study was to identify acute current stimulation effects by comparing ERG measurements before and during current stimulation. Therefore, the Wilcoxon signed-rank test was performed for the different ERG components (i.e., a-wave, b-wave, and b′-wave) and current applications (i.e., cathodal polarity, anodal polarity, and sham stimulation). Based on the multiple comparison problem of nine tests (i.e., three current applications with each three ERG components), the Bonferroni correction resulted in an adjusted significance value of *p*^∗^_*Wilcoxon*_≤ 0.0056. The effect strength was determined using the Cohens *d* value (Cohen, 1988).

The secondary aim of the study was to identify effects between the current applications on the ERG 2 measurement. Therefore, the Friedman test was performed for the ERG 2 measurement between the current applications with a Bonferroni correction of *p*^∗^_*Friedman*_≤ 0.016 (i.e., three tests, ERG 2 measurement with three ERG components). The ERG 1 measurement was performed equally for all groups before the current stimulation, so that it can be assumed that there is no difference between the groups.

For graphical analysis, grand mean signals for the three current applications and two ERG measurements over all volunteers were calculated. Furthermore, a violin plot for the graphical evaluation of data distribution was made. For this purpose, the difference between ERG 1 and ERG 2 measurements was calculated. Thus, a change around the value zero describes a change to higher or lower values for the ERG 2 measurement.

## Results

Electroretinogram signals could be derived and evaluated for all 15 subjects. Table 1 summarizes the mean ERG amplitudes and latencies averaged over all subjects.

**TABLE 1:** Measured mean values with standard deviation for the different current applications and measurements for both amplitudes and latencies.

Current application	Measurement	a-wave		b′-wave		b-wave	
		Amplitude in μV	Latency in ms	Amplitude in μV	Latency in ms	Amplitude in μV	Implicit time in ms
Cathodal	ERG 1	−22.407 ± 5.768	15.500 ± 1.278	36.160 ± 11.263	35.167 ± 1.386	58.567 ± 13.975	19.667 ± 0.943
	ERG 2	−22.178 ± 5.773	15.400 ± 1.114	37.165 ± 11.844	35.267 ± 1.365	59.342 ± 14.436	19.867 ± 0.785
Anodal	ERG 1	−21.209 ± 4.918	15.567 ± 0.981	36.915 ± 12.118	35.833 ± 1.660	58.124 ± 13.078	20.267 ± 1.078
	ERG 2	−21.121 ± 5.404	15.533 ± 1.040	36.746 ± 11.397	35.733 ± 1.289	57.867 ± 13.806	20.200 ± 1.046
Sham	ERG 1	−22.380 ± 5.290	15.367 ± 0.939	39.491 ± 10.996	35.567 ± 1.181	61.871 ± 13.030	20.200 ± 0.891
	ERG 2	−22.065 ± 4.759	15.533 ± 0.884	40.523 ± 12.095	35.700 ± 1.166	62.588 ± 13.825	20.167 ± 0.810

**FIGURE 2:**
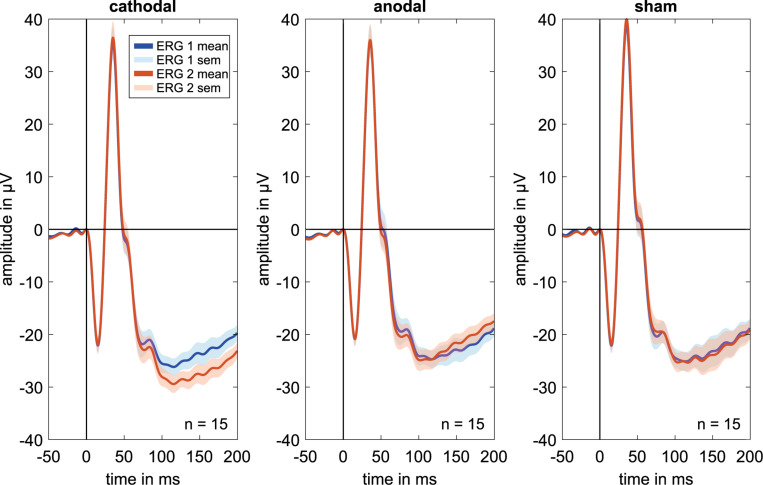
Grand mean signals for every stimulation group (i.e., cathodal polarity, anodal polarity, and sham stimulation; *n* = 15 for each curve) for the different ERG measurements. ERG 1 (blue curve) was done before and ERG 2 (orange curve) during the current stimulation. No effects are visible on the a, b′, or b-wave amplitudes or latencies comparing before and during current stimulation. Due to latency time differences between the subjects, different amplitudes could have occurred in the grand mean diagrams. Therefore, the grand mean signals show only a trend for the amplitude changes.

The ERG components indicated no clear effects in the comparison of ERG 1 to ERG 2. Figure 2 shows mostly overlapping grand mean signals over all volunteers for the different groups and measurements.

**FIGURE 3:**
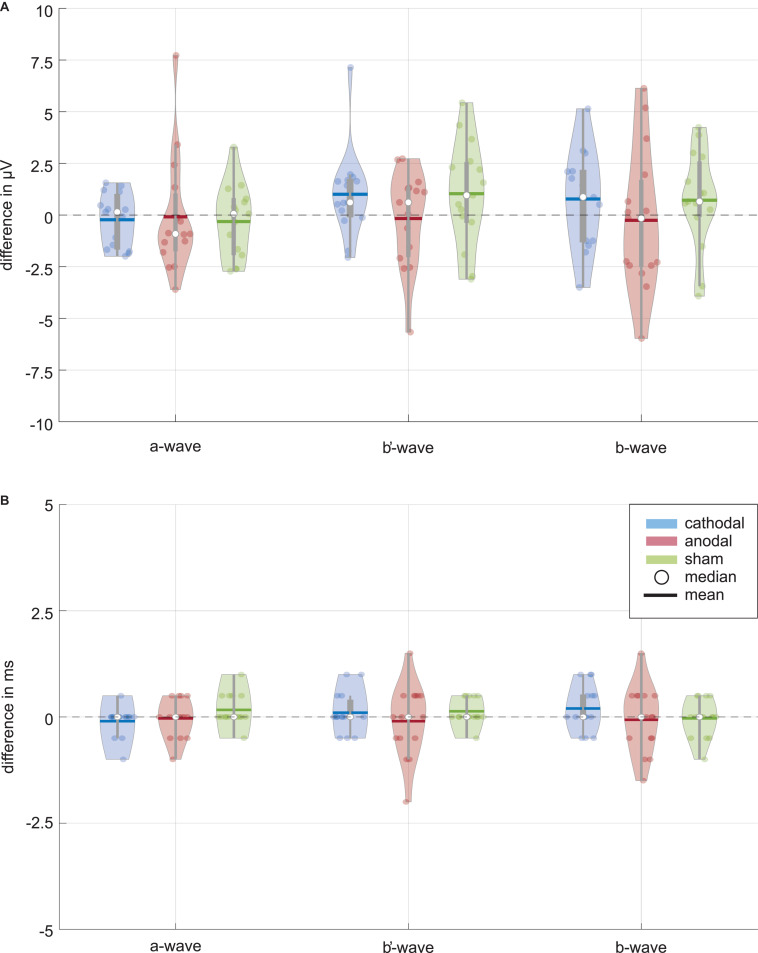
Data distribution of **(A)** amplitude and **(B)** latency differences between the ERG 1 and ERG 2 measurement for the ERG components (i.e., a-wave, b′-wave, and b-wave) and current stimulation groups (blue: cathodal polarity; red: anodal polarity; green: sham stimulation). The color-coded violin plots include data representations (*colored* dots) and the box-and-whisker plot (25 and 75% quartiles represented by the *gray* boxes and whiskers by the *gray* lines). Small changes are visible for the amplitudes of the cathodal and sham stimulation group while no changes can be seen for all latencies. Neither the Wilcoxon signed-rank test (α = 0.05, after Bonferroni correction p* ≤ 0.0056) comparing ERG 1 with ERG 2 measurement for the different amplitudes nor the Friedman test (α = 0.05, after Bonferroni correction p* ≤ 0.016) for the comparison between the current stimulation groups could find a significant current effect.

Figure 3A shows distributions of wave amplitude differences between ERG 1 and ERG 2. The a-wave amplitude decreased in the mean from ERG 1 to ERG 2 for all current stimulation groups. The cathodal and anodal stimulation group changed slightly by −0.23 ± 1.25 μV and −0.09 ± 2.75 μV, representing changes of −1.0% and −0.4%. For the sham stimulation group a mean change of −0.32 ± 1.69 μV (−1.4%) was found. The Wilcoxon signed-rank test could not find a significant difference between the two ERG measurements for all groups (*p*^∗^_*Wilcoxon*_ ≤ 0.0056; *p*_*cathodal*_ = 0.532, *p*_*anodal*_ = 0.427, *p*_*sham*_ = 0.570). The effect strength was for all stimulation groups < 0.1 (*d*_*cathodal*_ = 0.034, *d*_*anodal*_ = 0.019, *d*_*sham*_ = 0.059).

For the b′-wave the cathodal and sham stimulation group showed an increasing trend, while the anodal group showed a decreasing mean comparing before and during current stimulation (Figure 3). The cathodal and sham stimulation increased by 1.00 ± 2.03 μV (3.0%) and 1.03 ± 2.43 μV (2.6%), while the anodal current stimulation decreased slightly by −0.17 ± 2.24 μV (−0.5%). Also, for the b′-wave the Wilcoxon signed-rank test could not find a significant difference comparing the ERG 1 and ERG 2 measurement (*p*^∗^_*Wilcoxon*_ ≤ 0.0056; *p*_*cathodal*_ = 0.078, *p*_*anodal*_ = 0.955, *p*_*sham*_ = 0.140). The effect strength was for all stimulation groups < 0.1 (*d*_*cathodal*_ = 0.087, *d*_*anodal*_ = 0.017, *d*_*sham*_ = 0.086).

The b-wave, as sum of the a and b′-wave, showed for the cathodal and sham stimulation an increasing amplitude during the current stimulation (Figure 3). The amplitude changed in the mean by 0.78 ± 2.20 μV (1.3%) for the cathodal and 0.72 ± 2.28 μV (1.2%) for the sham stimulation group. In contrast, the anodal current stimulation group showed in the mean a decreasing change of −0.26 ± 3.26 μV, which corresponds to a percentage change of −0.4%. Again, no significant difference between the ERG 1 and ERG 2 measurement could be found for all groups (*p*^∗^_*Wilcoxon*_ ≤ 0.0056; *p*_*cathodal*_ = 0.233, *p*_*anodal*_ = 0.691, *p*_*sham*_ = 0.156). Table 2 summarizes the Wilcoxon signed-rank test results for the primary aim of the study. The effect strength was for all stimulation groups <0.1 (*d*_*cathodal*_ = 0.05, *d*_*anodal*_ = 0.015, *d*_*sham*_ = 0.052).

**TABLE 2:** Wilcoxon signed-rank test *p*-values, whether there is amplitude difference between the baseline measurement and the ERG measurement during the current stimulation.

Current stimulation application	Amplitude	*p*-value
Cathodal polarity	a-wave	0.532
	b′-wave	0.078
	b-wave	0.233
Anodal polarity	a-wave	0.427
	b′-wave	0.955
	b-wave	0.691
Sham stimulation	a-wave	0.570
	b′-wave	0.140
	b-wave	0.156

The significance level was set to α = 0.05 and, after Bonferroni correction, the adjusted significance value was p*_Wilcoxon_≤ 0.0056.

ERG wave latencies indicated no common current effects, as shown in Figure 3B, whereupon a statistical evaluation of the latency differences was waived.

As to the secondary aim of the study, the current stimulation groups should be tested for significant differences between them for the ERG 2 measurement. The data distribution analysis in Figure 3 showed a trend for differences, especially for the b-wave between the anodal and sham stimulation group. The Friedman test could not find a significant difference for the ERG 2 measurement between the cathodal, anodal and sham stimulation group for all ERG components (*p*^∗^_*Friedman*_ ≤ 0.016; *p*_*a–wave*_ = 0.189, *p*_*b*′–wave_ = 0.085, *p*_*b–wave*_ = 0.041, cf. Table 3).

**TABLE 3:** *P*-values for Friedman test, whether there is a difference between the three current stimulation groups (i.e., cathodal polarity, anodal polarity, and sham stimulation) for the characteristic full field ERG amplitudes during the current stimulation (ERG 2).

Amplitude	Measurement	*p*-value
a-wave	ERG 2	0.189
b‘-wave	ERG 2	0.085
b-wave	ERG 2	0.041

The ERG 1 measurement was performed equally for all groups before the current stimulation, so that it can be assumed that there is no difference. The significance level was set to α = 0.05 and, after Bonferroni correction, the adjusted significance value was p*_Friedman_≤ 0.016.

## Discussion

In this present study, the authors aimed to test whether or not full field ERG components mainly generated by pre-ganglion cells demonstrate acute effects to ocular direct current stimulation. Therefore, the researchers stimulated 15 subjects in three independent sessions, each with another current application (i.e., cathodal polarity, anodal polarity or sham stimulation). The authors could determine no current stimulation effects on the amplitudes or latencies in the evaluated ERG waves. Furthermore, they could find no significant difference across the three current applications during the current stimulation.

In the preceding PERG study, Blum et al. (2020) found a significant difference for the P50 amplitude for both anodal and cathodal current stimulation. The P50 amplitude is composed of the activity of ganglion cells and, to a small extent, of the activity of pre-ganglion cells. Whether the current effect results from the influence of ganglion cells or pre-ganglion cells remained unknown. In this study, the authors investigated current stimulation effects on the light-adapted full field ERG. The origin of the ERGs a- and b-wave can be traced back to pre-ganglion cells, especially the cones and bipolar cells. Here, we did not find a significant current effect on the a- or b-wave during the applied current stimulation. Therefore, it can be assumed that the pre-ganglion cells did not contribute to the significant effect on the P50 amplitude in the PERG study for the applied current stimulation setup. Since no current stimulation effects were detected in the present study, hypothesized polarity-dependent current stimulation effects on pre-ganglion cells could not be evaluated.

Limitations of the study lie in the current stimulation parameters: the electrode positions, current strength, and stimulation time, as well as the number of participants and the study design. Based on our preceding study indicating stimulation effects on PERG (Blum et al., 2020), we expected an effect size of *d* = 0.9. Under this condition, the present study has a power of (1−β) = 0.94. However, the calculated effect sizes within the present study are all <0.1, which is a strong indication for the absence of an effect of the here applied stimulation on pre-ganglion cells. Furthermore, in this study and in the PERG study, the authors used the same electrode positions for current stimulation and ERG recording. Current stimulation effects depend on the neuronal morphology relative to the generated electric field (Bikson et al., 2019). Therefore, the generated electric field by the positions of the current stimulation electrodes might be better suited for stimulating ganglion cells and their axons than for bipolar cells and cones. It cannot be excluded that a repositioning of the return electrode from the ipsilateral temple to another position could influence the bipolar cells and cones. For example, a positioning at the back of the head to the Oz position (after 10–20 system) would generate a more homogeneous current flow through the entire eye (Hunold et al., 2015) and could therefore produce other effects. Furthermore, in both studies the eye was not stimulated consistently because of both the cutout in the ring electrode for ERG recording at the lower eyelid and the position of the return electrode. A more homogeneous stimulation of the eye could generate other results. In addition, the authors stimulated with a current strength of 800 μA for 5 min to maintain a low current load. Higher current intensities and longer stimulation duration can have different effects (Jamil et al., 2017). Furthermore, the authors did not investigate after-effects of the current stimulation. Studies combining transcranial direct current stimulation with VEPs showed that after-effects can occur (Antal et al., 2004; Wunder et al., 2018). However, the study design of this research as well as the design of the preceding PERG study refer to the effects during current stimulation. Due to the statistical multiple test problem, own study designs should be developed for the evaluation of after-effects.

For all stimulation groups, it is visible that the amplitudes of both measurements get negative after the b-wave at about 100 ms in the grand mean figure (Figure 2). In the anodal and especially in the cathodal stimulation groups, an increasing negative amplitude course can be seen for the ERG 2 measurement. Another stimulation setup should be used to investigate effects on this negative course. The negative amplitude is most distinctive for a light-adapted full field ERG with a brief red flash (≤5 ms) on a blue background. Under these conditions, a more defined negative amplitude named photopic negative response (PhNR) occurs after the b-wave at about 70 ms, which can be attributed to the additional response of ganglion cells and their axons to the light stimulus (Rangaswamy et al., 2007; Machida, 2012; Frishman et al., 2018). In this study, the PhNR amplitude was not recorded in conformity with ISCEV standard (Frishman et al., 2018) and was therefore not included in the study analysis. A further investigation of the PhNR amplitude after ISCEV-compliant recording could provide valuable information about the current effect on middle and outer retinal layers. The high number of parameters that can be evaluated in such study requires a special study design to counteract the multiple test problem as much as possible.

## Conclusion

With regard to the stimulation design in this study, no amplitude or latency changes occur for the full field ERG during an ocular direct current stimulation. Furthermore, no differences between the stimulation groups could be found. These results support the hypothesis that the known current effects for the PERG are due to the influence of ganglion cells and not of pre-ganglion cells. The investigation of a full field ERG standardized for the PhNR amplitude could provide valuable information, because the function of middle and outer retinal layers can be evaluated simultaneously.

## Data Availability Statement

The datasets generated for this study are available on request to the corresponding author.

## Ethics Statement

The studies involving human participants were reviewed and approved by Ethics commission at the medical faculty of the Friedrich-Schiller-University Jena. The patients/participants provided their written informed consent to participate in this study.

## Author Contributions

M-CB: conceptualization, methodology, data acquisition and curation, data processing and analysis, manuscript drafting, and manuscript revision. AH and BS: conceptualization, methodology, and manuscript revision. SK: project administration and supervision, conceptualization, methodology, and manuscript revision. All authors contributed to the article and approved the submitted version.

## Conflict of Interest

The authors declare that the research was conducted in the absence of any commercial or financial relationships that could be construed as a potential conflict of interest.
